# Indigenous knowledge systems and pandemic health resilience

**DOI:** 10.3389/fpubh.2026.1853958

**Published:** 2026-08-03

**Authors:** Wilfred Lunga, Charles Musarurwa, Mkhokheli Sithole, Olivia Kunguma, Mildred Ziweya, Caiphus Baloyi

**Affiliations:** 1 Disaster Management Training and Education Centre for Africa, University of the Free State, Bloemfontein, South Africa; 2 Human Sciences Research Council, Pretoria, South Africa; 3 Northwest University Africa Centre for Disaster Studies, Potchefstroom, South Africa; 4 Department of Science, Technology and Design Education, Faculty of Education, Midlands State University, Gweru, Zimbabwe; 5 Faculty of Business and Economic Sciences, Institute of Development Sciences, National University of Science and Technology, Bulawayo, Zimbabwe; 6 Department of Curriculum and Instruction, Faculty of Humanities, Education and Social Sciences, University of Botswana, Gaborone, Botswana; 7 School of Social Science, College of Humanities, University of KwaZulu-Natal, Durban, South Africa

**Keywords:** BRICS, COVID-19, immune health, indigenous knowledge systems, pandemic preparedness, perceived effectiveness, planetary health, respiratory health

## Abstract

**Introduction:**

Indigenous Knowledge Systems (IKS) have played important roles in community-based health responses across the Global South, yet they remain marginalized in formal health policy during global health crises. This study examined how IKS were mobilized to support respiratory and immune health during the COVID-19 pandemic across BRICS (Brazil, Russia, India, China, South Africa) and Southern African Development Community (SADC) countries.

**Methods:**

A qualitative exploratory research design was employed across Zimbabwe, South Africa, and China involving 38 participants. Data were collected through semi-structured interviews (n = 22), digital questionnaires (n = 10), and WhatsApp voice narratives (n = 6). Purposive and criterion-based sampling ensured representation across age, gender, geography, and familiarity with IKS. Data were analysed using Braun and Clarke’s six-phase thematic analysis with inter-rater reliability verification (Cohen’s κ = 0.82). Member checking was conducted through community advisory boards, and ethical approval was obtained from institutional review boards in each participating country.

**Results:**

Participants reported using 27 distinct herbal remedies, primarily derived from locally available plants, including *Lippia javanica, Artemisia afra, Psidium guajava*, and commonly used spices, with perceived improvements in respiratory symptoms and overall well-being. Four major themes emerged: (1) variation in institutional support, with China and India formally integrating traditional medicine while SADC countries relied largely on informal community practices; (2) differences in knowledge documentation, characterized by codified systems in China and India compared with predominantly oral transmission in SADC countries; (3) barriers to integration, including epistemic hierarchies, regulatory limitations, and policy marginalization; and (4) community resilience and adaptability despite limited formal recognition and support.

**Discussion:**

The findings suggest that IKS can complement biomedical approaches in strengthening health system resilience during public health emergencies. Integrating IKS into pandemic preparedness and response requires policies that recognize epistemological diversity, protect intellectual property, promote equitable benefit-sharing, and strengthen community knowledge sovereignty within planetary health frameworks.

## 1 Introduction

### 1.1 Indigenous knowledge systems: definition and context

Indigenous Knowledge (IK) encompasses long-standing traditions, skills, and philosophies developed by Indigenous people through sustained interaction with their natural and social environments (1, 2). These systems are cumulative, dynamic, context-specific, orally transmitted, and non-formal, coexisting alongside formal scientific knowledge even in urban settings (3). Far from static, IK continuously evolves, reflecting the lived realities of Indigenous communities globally (2). Populations across Africa, the Americas, Asia, and Oceania have cultivated rich epistemologies rooted in historical, spiritual, and ecological worldviews (4, 5).

In Africa, Indigenous Knowledge has long been central to addressing health and environmental challenges (6–8). Traditional medicine remains the first line of care for many, offering holistic approaches to prevention and treatment of disease (9, 10). African communities have for generations relied on herbal and traditional remedies to manage human and animal health (11–13). These practices emphasize balance across physical, emotional, and spiritual dimensions of health, an approach increasingly recognized as vital during global health crises when conventional healthcare systems may be overstretched or inaccessible (14, 15).

During the COVID-19 pandemic, traditional health practitioners in Africa and parts of Asia played key roles in community-based care, promoting immune-supportive herbs and respiratory therapies (16–18). Despite this engagement, colonial legacies and dominant biomedical paradigms have historically delegitimised these systems, reducing their perceived legitimacy in formal health policies and practice (19–21).

### 1.2 Global health crises and the renewed recognition of IKS

Recent global health emergencies have prompted renewed recognition of IK in strengthening health resilience, particularly among vulnerable and underserved populations. Framing IK within a planetary health perspective highlights the intrinsic links between human well-being and ecosystem integrity, acknowledging that ecological balance, community resilience, and cultural coherence are inseparable (22–25). Integrating IK into health systems and emergency preparedness can improve equity, access, and resilience whilst simultaneously supporting sustainable environmental stewardship, aligning health interventions with the broader goals of planetary health (15, 24).

South Africa, as a member of both SADC and BRICS, provides a unique context to study how IK is mobilized and adapted in response to respiratory and immune health threats, reflecting the interplay of cultural diversity, migration, and global health challenges (26). This study examines the concurrent application of modern biomedical approaches and Indigenous Knowledge Systems (IKS) in enhancing respiratory and immune health responses during global health crises, drawing from experiences of selected BRICS and SADC countries (Zimbabwe, South Africa, and China).

### 1.3 Epistemological marginalization of IKS and Decolonial imperatives

Historically, IKS has been marginalized due to its oral transmission, local linguistic frameworks, and ontologies that often diverge from Euro-Western empiricism (1, 3). IKS represents an epistemic system that is not only scientific but also adaptive and contextually situated, evolving in response to changing socio-ecological realities (3). However, colonial legacies have institutionalized Western medicine as the gold standard whilst framing traditional practices as ‘unscientific’ or ‘superstitious’ (19). In SADC nations, restrictive colonial-era policies such as the Witchcraft Suppression Act continue to criminalize traditional healing, undermining opportunities for integrated healthcare approaches (27). In Zimbabwe, the Witchcraft Suppression Act was first enacted in 1899 to suppress traditional practices but was later amended in 2006 to acknowledge the existence of traditional medicine, though the core provisions remained substantially unchanged (27).

Despite this, recent policy shifts and scholarly attention have begun to re-evaluate the utility of IKS, especially in the face of global health emergencies where biomedical solutions may be delayed, inaccessible, or inadequate (4, 17).

### 1.4 Research gap and study objectives

Whilst individual studies document IKS use in specific countries, comparative analysis of how diverse epistemological systems (oral African traditions versus documented Chinese traditions) operate within pandemic responses remains limited. Thus, this study addresses this gap by examining traditional medicine mobilization across geographically and epistemologically diverse BRICS and SADC contexts.

### 1.5 Research questions

*RQ1* How did individuals in BRICS and SADC countries incorporate traditional practices into preventing and managing respiratory and immune-related health challenges during COVID-19?

*RQ2* What barriers and opportunities shaped access to and use of Indigenous Knowledge Systems during the pandemic?

*RQ3* How do formal institutional responses to IKS differ across BRICS countries (China, India) compare with SADC countries (Zimbabwe, South Africa)?

*RQ4* How did digital platforms facilitate the dissemination of Indigenous knowledge during global health crises?

## 2 Literature review

### 2.1 Indigenous knowledge systems and traditional medicine as primary healthcare

Global health crises such as the COVID-19 pandemic have amplified the need for inclusive, context-sensitive, and holistic health systems that recognize and integrate diverse epistemologies (1, 3). IKS, anchored in local experiences, ecological familiarity, and socio-cultural practices, offer rich, underexplored potential in supporting immune and respiratory health, especially in regions with fragile biomedical infrastructures (28, 29).

Indigenous medical systems, often rooted in ethnobotany and holistic wellness paradigms, have long functioned as primary healthcare modalities across much of the Global South (30, 31). According to UNESCO (10), approximately 80% of the African population continues to rely on traditional medicine, a trend mirrored in India and China, where Ayurveda and Traditional Chinese Medicine (TCM) are deeply institutionalized (32). These systems emphasize preventive care, immune modulation, and restoration of bodily harmony principles that are now being increasingly validated by biomedical research (30, 33).

### 2.2 Scientific validation of traditional herbal remedies

Scientific investigations have validated the antiviral, anti-inflammatory, and immunomodulatory effects of many traditional herbs used in African and Asian IKS (13, 31). For instance, *Artemisia afra*, *Hypoxis hemerocallidea* (African potato), and *Pelargonium sidoides* have shown pharmacological efficacy in treating respiratory tract infections (13, 34, 35). Empirical evidence also supports the health benefits of plant-based, whole-food diets common in Indigenous communities. Kim et al. (30) found that individuals on predominantly plant-based diets experienced less severe health symptoms, likely due to improved immune function. This corroborates long-standing indigenous dietary practices that incorporate nutrient-dense, phytochemical-rich foods and herbs (36).

Furthermore, Heiat et al. (33) estimate that 25% of modern pharmaceuticals are derived from traditional remedies, underscoring the deep scientific value embedded in IKS. These include compounds from medicinal plants such as *Catharanthus roseus* (source of vincristine) and *Salix alba* (source of aspirin), which originated from indigenous medical traditions and were later standardized for global use (37). However, it is worth noting that the digital revolution has played a pivotal role in reshaping access to IKS. During the COVID-19 pandemic, the dissemination of traditional remedies via social media platforms such as WhatsApp and Facebook catalyzed a new wave of ethnobotanical engagement, both within and beyond indigenous communities (8, 38). Whilst concerns remain about misinformation, these platforms democratized access to knowledge that was once restricted to specific lineages and oral networks (38).

### 2.3 Comparative integration across BRICS and SADC nations

Among BRICS countries, China provides a robust model for integrating traditional and modern health systems. Traditional Chinese Medicine (TCM), with documented roots dating back over two millennia, was used extensively during the COVID-19 pandemic (39). State-sanctioned protocols included herbal formulations such as Lianhuaqingwen and Jinhuaqinggan for mild cases and Xuebijing for severe symptoms (39, 40). Clinical trials further validated the efficacy of these treatments, prompting their inclusion in the national health response (41, 42).

India’s Ayurvedic sector also responded proactively, with the Ministry of AYUSH issuing guidelines for immunity-boosting regimes (43, 44). However, unlike China, India’s fragmented healthcare governance and limited documentation of clinical outcomes have hampered wider integration (32, 45). In contrast, Brazil’s pandemic response largely overlooked the wisdom embedded within the Amazon’s indigenous populations (46). Despite their use of herbal disinfectants and isolation practices, these communities received minimal formal support or recognition from federal authorities. South Africa, whilst home to rich ethnomedical traditions, continues to experience tension between biomedical and indigenous healing systems (35, 47). The COVID-19 pandemic, however, prompted renewed engagement between state structures and traditional healers, though this has yet to translate into policy coherence or integration (15).

## 3 Methods and materials

### 3.1 Research design and philosophical positioning

This study employed a qualitative exploratory research design using purposive and criterion-based sampling to investigate how Indigenous Knowledge Systems (IKS) were mobilized to respond to COVID-19 in BRICS and SADC contexts (48, 49). Qualitative approaches are suited to examining culturally situated phenomena and non-quantifiable health practices, especially when research aims to centre local epistemologies rather than impose external frameworks (50, 51). This approach aligns with decolonial research principles advocating for indigenous-centred methodologies (49, 52). Two theoretical frameworks guided the research, these being Planetary Health and the Decolonial paradigm. Planetary health recognizes the interconnections between human health, ecosystem integrity, and social justice. It recognizes that pandemic responses must address disease containment, environmental stewardship, and equity (24, 25). The decolonial framework acknowledges Western biomedical paradigm as one epistemology among many, not a universal truth. It seeks to validate local knowledge systems and their epistemic authority (2, 49, 53).

**Figure 1:**
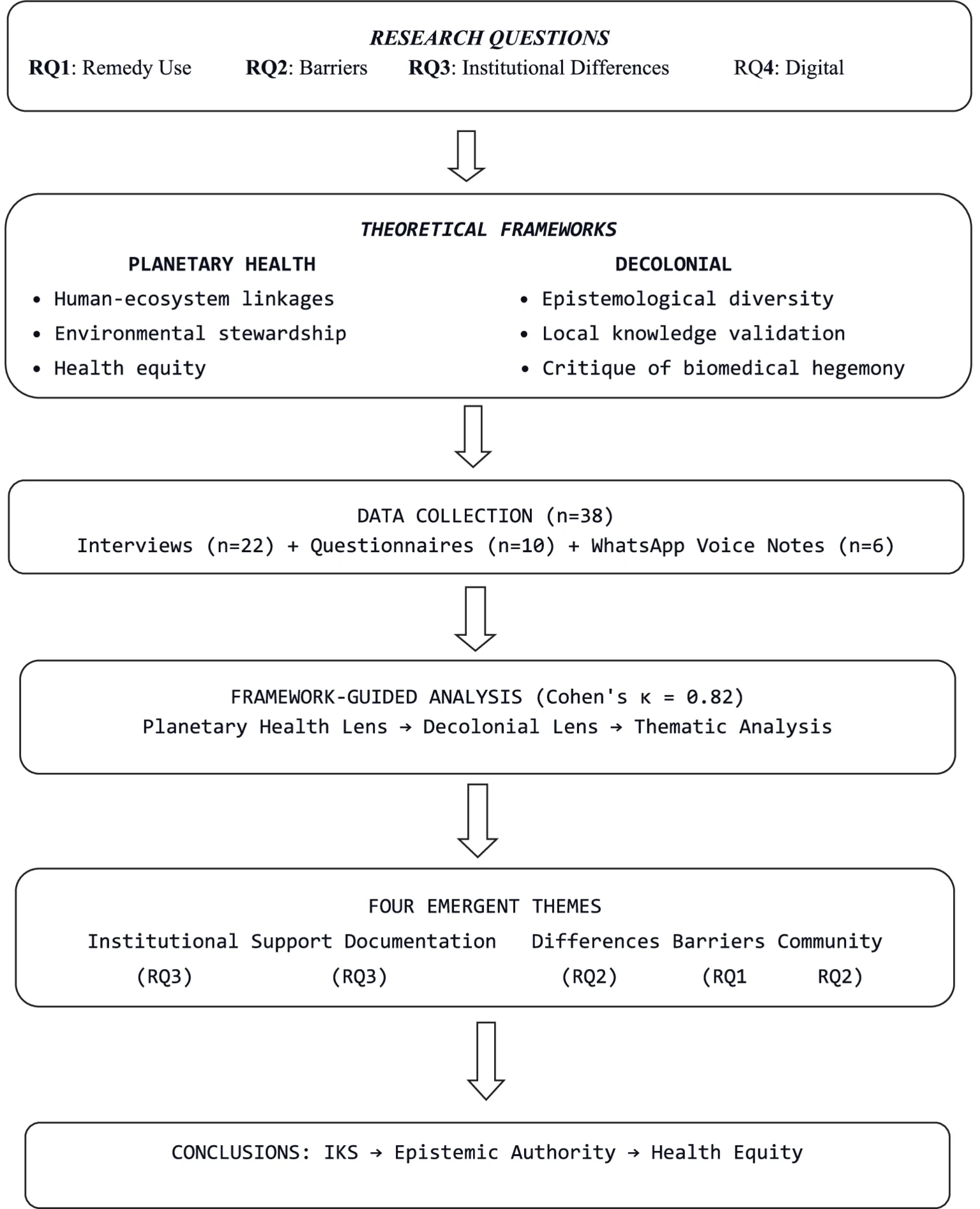
Theoretical Framework Integration – Conceptual Diagram. *Integration of planetary health and decolonial frameworks guiding data collection (*n* = 38), thematic analysis (Cohen’s *κ* = 0.82), and emergent themes. IKS, Indigenous Knowledge Systems.

Figure 1 illustrates how these frameworks were integrated with data collection, analysis, and interpretation.

### 3.2 Application of the planetary health and decolonial frameworks to data collection and analysis

The planetary health framework (24, 25) was operationalized throughout data collection by designing interview questions that explicitly probed participants’ use of locally available ecological resources (e.g., “Which plants did you harvest from your immediate environment?”). Questions further explored reciprocal relationships between environmental stewardship and health practices (e.g., “How do you ensure sustainable harvesting of these remedies?”), while the interview guide incorporated structured probes regarding community-level resilience strategies that directly address health–environment–equity linkages (25). During data analysis, the coding scheme was constructed *a priori* to include nodes specifically for “ecosystem resource use,” “environmental stewardship practices,” and “community resilience strategies” (24). Emergent themes were systematically evaluated for their relevance to planetary health principles, particularly concerning sustainable resource extraction and health equity, such that interpretations explicitly considered how mobilization of indigenous and local knowledge systems simultaneously addressed the health, environmental, and equity dimensions of crisis response (24, 25).

Concurrently, the decolonial framework (2, 49, 53) was operationalized by centring participant perspectives rather than imposing external biomedical frameworks during data collection. For example, questions were phrased as “How did you know this remedy was working?” rather than “What clinical improvements did you measure?” thereby honoring non-biomedical conceptualisations of health and effectiveness, including spiritual and holistic dimensions, while the interview guide maintained a clear distinction between oral knowledge traditions and documented systems without value judgments (2, 49). In the analytic phase, the coding scheme explicitly examined epistemological differences between documented traditions (e.g., BRICS contexts) and oral traditions (e.g., SADC contexts), with analytical memos systematically documenting instances where biomedical frameworks would have excluded participant perspectives (53). Themes were developed with sustained attention to power dynamics within knowledge hierarchies, particularly regarding which knowledge systems are historically considered “valid,” and negative case analysis was deliberately extended to include dissenting perspectives that challenged both biomedical and traditional paradigms, thereby addressing reviewer concerns regarding epistemic reflexivity (2, 53).

### 3.3 Research setting and country selection rationale

Three countries were selected based on the following rationale (Table 1).

**Table 1:** Research setting and country selection rationale.

Country	Selection rationale	Key characteristics
Zimbabwe	African oral-based IKS traditions and the SADC member	Limited formal healthcare infrastructure; high reliance on traditional medicine; under-resourced health system during COVID-19 (27)
South Africa	BRICS and the SADC member	Significant traditional healer population; formalized traditional health practitioner regulations (Traditional Health Practitioners Act, 2007); tensions between biomedical and traditional systems (35)
China	BRICS member	Formalized Traditional Chinese Medicine (TCM) with government backing; documented herbal traditions; state-integrated pandemic response (60, 70)

### 3.4 Participant recruitment and sampling

#### 3.4.1 Sampling strategy and inclusion criteria

A purposive sampling approach was employed to recruit participants across three focus countries (48, 49). Purposive sampling enables targeted recruitment of individuals meeting specific criteria known to illuminate the research question (54).

Eligibility for participation was determined using a structured set of inclusion and exclusion criteria aligned with the study’s planetary health and decolonial frameworks (25, 53). Inclusion required participants to be aged 18 years or older, to report either a confirmed or clinically suspected diagnosis of COVID-19 during the period March 2020 to December 2021, and to have documented use of at least one traditional or indigenous remedy during their illness. Additionally, participants were required to provide voluntary informed consent and to communicate competently in English, Shona, Zulu, or Mandarin, thereby respecting linguistic diversity and avoiding the exclusionary effects of monolingual research designs (49). Exclusion criteria comprised hospitalization requiring intensive care, as severe disease trajectories were considered likely to preclude reliable retrospective recall of remedy use and environmental resource access, and cognitive impairment of any etiology that would compromise the ability to provide informed consent (2). These criteria were deliberately designed neither to privilege biomedical over indigenous conceptualisations of illness severity nor to systematically exclude oral knowledge traditions, consistent with decolonial commitments to epistemic reflexivity (24, 53).

#### 3.4.2 Recruitment procedures

Participants were recruited through (1): community health worker networks in rural areas (2); digital platforms (WhatsApp groups, Facebook pages); and (3) snowball referrals (54). This mixed approach enabled geographic and socioeconomic diversity. Recruitment occurred between June 2021 and March 2022, approximately 3–6 months post-acute illness.

#### 3.4.3 Sample composition and justification

The relatively small sample (*n* = 38) limits statistical generalisability; however, it is appropriate for qualitative inquiry where the objective is depth rather than breadth (48, 55). Evidence suggests that smaller samples can achieve data saturation, particularly when participants share relevant experiences and the research design is focused (48, 55). For qualitative exploratory research, adequacy is determined by data saturation, the point at which additional data collection yields no new thematic insights (54). Thematic saturation occurred at approximately 30 interviews; data collection continued to 38 to ensure country representation and demographic diversity (Table 2; Table 3).

**Table 2:** Sample composition and justification.

Country	Sample Size (n)	Percentage
Zimbabwe	14	36.8%
South Africa	16	42.1%
China	8	21.1%
Total	38	100%

**Table 3:** Data collection methods and characteristics.

Method	Sample size (*n*, %)	Mode of administration	Duration	Instrument	Data capture
Semi-structured interviews	22 (57.9%)	Virtual via Zoom or telephone	45–90 min	Standardized guide with 24 questions across 7 domains	Audio-recorded with permission
Open-ended digital questionnaires	10 (26.3%)	Google Forms	20–40 min	Questions mirrored the interview guide	Digital capture via a platform
WhatsApp voice notes	6 (15.8%)	WhatsApp (low-connectivity contexts)	Variable (participant-led)	Narrative reflections guided by key themes	Voice notes transcribed verbatim

### 3.5 Data collection methods

#### 3.5.1 Multi-modal data collection

##### 3.5.1.1 Data collection instrument development

The semi-structured interview guide was developed through literature review, consultation with community advisory boards, pilot testing with 5 participants, and iterative refinement based on feedback (48, 49). The research team received standardized training including: a 3-day workshop on qualitative interview techniques, research ethics, cultural sensitivity, and data security; competency assessment through mock interviews; and ongoing supervision and quality monitoring (48).

##### 3.5.1.2 Data management and security

All data handling procedures complied with GDPR and applicable national data protection requirements (25). Participant anonymity was ensured by assigning unique numeric codes, with all direct identifiers removed before analysis. Data was stored in AES-256-encrypted databases, and role-based access controls restricted data visibility to authorized personnel only (2). Original audio files were permanently deleted immediately following transcription verification, thereby minimizing prolonged exposure of potentially identifiable raw data (49). Identifiable data, including consent forms and code-linking logs, were scheduled for destruction 1 year after data collection completion. In contrast, fully de-identified datasets were retained for 5 years to enable secondary verification and reproducibility, balancing scientific transparency with the ethical imperative to avoid extractive data custody over indigenous knowledge systems (24, 53).

##### 3.5.1.3 Translation and quality assurance

Interviews in Shona, Zulu, and Mandarin were translated into English by bilingual researchers (48, 56). Quality assurance procedures included back-translation (15% of interviews), semantic equivalence verification, and research team consensus meetings (57).

### 3.6 Data analysis

Thematic analysis was conducted following the six-phase approach outlined by Braun and Clarke (48) (Table 4).

**Table 4:** Thematic analysis was conducted following the six-phase approach outlined by Braun and Clarke (48).

Phase	Activity	Description
Phase 1	Familiarization	Research team read all transcripts multiple times; created initial analytical memos
Phase 2	Systematic coding	All transcripts coded independently by two researchers; 20% (8 interviews) coded by both; Cohen’s kappa = 0.82 (substantial agreement)
Phase 3	Theme development	Coded segments organized into preliminary themes; refined through iterative team discussion
Phase 4	Review and refinement	Themes checked against raw data; negative case analysis conducted; audit trail maintained
Phase 5	Definition and naming	Final themes defined with clear conceptual boundaries; exemplary quotes identified
Phase 6	Report production	Themes organized into a coherent narrative; findings contextualized within existing literature

Analysis was supported by ATLASti qualitative analysis software, which facilitated systematic data organization and descriptive statistics while maintaining a comprehensive audit trail as recommended by Denzin (54).

### 3.7 Trustworthiness strategies

Methodological rigor was established through triangulation of multiple data sources and collection methods, enabling cross-verification of emergent findings across participant accounts and contextual datasets (48, 54). Member checking was conducted by presenting preliminary thematic findings to 12 community advisory board members (three per country: South Africa, Zimbabwe, and China), and their feedback was systematically incorporated to refine theme interpretations, thereby enhancing descriptive and interpretive validity (58).

Reflexivity was operationalised through research team members maintaining reflective journals that documented positionality, cultural assumptions, and potential biases throughout the research process, with regular team discussions of journal entries to surface and address epistemic blind spots (48). Peer debriefing involved team members not involved in initial coding independently reviewing a subset of coded transcripts and analytic memos, after which critical feedback was discussed and adjudicated in consensus meetings, further strengthening analytic rigor (54).

### 3.8 Ethical considerations

Ethical approval was secured from multiple institutional review bodies: Midlands State University Research Ethics Committee (Zimbabwe), Human Sciences Research Council COVID-19 Ethics Protocol covering BRICS countries. Informed consent was digitally obtained using translated standardized forms (48, 49). Strict confidentiality was maintained, with all identifying information replaced by participant codes (56). A comprehensive duty of care protocol was implemented, including mental health resources for participants who experienced distress, and the research team received specialized training in trauma-informed care protocols (48). Community advisory boards were actively involved from design through dissemination (49).

Methodological Limitations and Implications for Interpretation whilst this study provides rich qualitative insights grounded in rigorous thematic analysis (Cohen’s *κ* = 0.82), several methodological limitations warrant transparent acknowledgment and must inform interpretation of findings. First, the small sample size (*n* = 38), whilst not statistically representative, proved adequate for achieving qualitative thematic saturation in exploratory research (48, 55). However, this adequacy applies specifically to the three countries studied; generalization beyond Zimbabwe, South Africa, and China to the full BRICS and SADC regions remains inappropriate (58). Notably, Brazil and Russia, two BRICS members, are absent from this analysis, and regional variation within the three sampled countries was not systematically explored, limiting our ability to capture the heterogeneity of IKS integration across these geographically and culturally diverse contexts.

Second, the hybrid purposive-plus-snowball sampling approach, whilst justified by pandemic-related recruitment constraints, introduced potential selection bias. Individuals with more positive views and experiences of traditional remedies were more likely to be recruited through community networks and WhatsApp groups, potentially favoring success narratives. Those with negative experiences, failed remedy use, or skepticism toward IKS are systematically underrepresented (59). This selection bias likely inflates the perceived effectiveness estimate and may obscure important counterfactuals and safety concerns.

Third, reliance on self-reported outcomes with no objective clinical measures meant that placebo effects could not be ruled out (60, 61). The study documents subjective symptom improvement and perceived benefit, not clinical efficacy validated through biomedical testing. No clinical trials were conducted to validate specific remedies used; the ethnopharmacological evidence cited in our results represents external validation from published literature, not primary evidence from our cohort. While eight participants (21.1%) reported minor adverse effects (nausea, stomach upset, allergic reactions, diarrhea), the absence of serious adverse events does not constitute a rigorous safety assessment. Pharmacovigilance systems and standardized adverse event monitoring would be required for comprehensive safety profiling.

Fourth, temporality introduces recall bias. Data collection occurred 3–12 months post-illness, introducing potential temporal distance effects on recall accuracy (62). Participants’ memories of specific remedies used, their sequencing, duration, and perceived effectiveness may have been reshaped by subsequent health experiences, community narratives, or media discourse. This temporal gap particularly affects precision regarding remedy attribution for symptom improvement.

Fifth, translation processes between English, Shona, Zulu, and Mandarin may have introduced subtle shifts in meaning, particularly regarding concepts of health, effectiveness, and spiritual dimensions of healing that may not map straightforwardly across epistemological systems. Although back-translation procedures were conducted on 15% of interview transcripts (56, 57), the cultural specificity of health concepts may still have been attenuated through the translation process. Non-standardized language items and culturally embedded metaphors risk homogenization when converted to English-centric analytical frameworks.

Sixth, COVID-19 illness severity was self-reported, with 84.2% of participants characterizing their illness as mild and 15.8% as moderate. No participants with severe illness requiring intensive care were included per study exclusion criteria. This exclusion, whilst justified on methodological grounds (inability to provide retrospective recall), means we have no data on how IKS were utilized or how they failed in severe illness contexts.

The findings thus represent responses to predominantly mild-to-moderate disease, with unknown generalizability to critical illness trajectories. These limitations collectively suggest that whilst this study documents important community health practices, institutional variation, and policy gaps, caution is warranted regarding effect size estimates and generalization. The findings should be interpreted as exploratory evidence about how communities mobilize indigenous knowledge, not as proof of clinical efficacy.

## 4 Results

### 4.1 Participant characteristics

The sample comprised participants across three countries (South Africa, Zimbabwe, and China). Descriptive statistics reveal that the mean age was 52.4 years (SD ±15.3), with 55.3% of participants identifying as female. Educational attainment varied, with 42.1% having completed secondary school as their highest level of education, while the majority (71.1%) resided in urban or peri-urban settings. Prior familiarity with indigenous and local knowledge systems (IKS) through family history was reported by 68.4% of participants.

Regarding COVID-19 illness severity, 84.2% of participants rated their illness as mild, while 15.8% characterized their symptoms as moderate; no participants with severe illness requiring intensive care were included per exclusion criteria. Notably, 84.2% of participants reported that they did not consult any formal healthcare provider during their illness, citing cost and accessibility barriers as the primary reasons for non-consultation. Twenty-seven distinct remedies were documented. Overall perceived effectiveness was very high across all remedy instances. Critically, this perception of effectiveness represents participants’ subjective experience of symptom improvement, not clinical efficacy. Place effects, natural disease courses, and concurrent biomedical treatments cannot be ruled out as contributors to reported improvement (Table 5).

**Table 5:** Most frequently used remedies and perceived helpfulness.

Remedy	Common name	Scientific name	Primary use
Zumbani	Lemon bush	*Lippia javanica*	Respiratory symptoms
Mhlonyane	African wormwood	*Artemisia afra*	Cough, congestion
Guava leaves	Guava	*Psidium guajava*	Fever, cough
Honey	Honey	*Apis mellifera*	Sore throat, cough
Ginger	Ginger	*Zingiber officinale*	Immune support
Garlic	Garlic	*Allium sativum*	Immune support

These findings align with ethnopharmacological research demonstrating the therapeutic properties of the documented plants. Artemisia afra has been traditionally used for respiratory conditions and has shown antiviral properties in preliminary studies (35, 63). Lippia javanica has long been employed for colds and respiratory infections and possesses documented antimicrobial properties (28, 64). *Psidium guajava* leaves have been traditionally used to manage fever and respiratory symptoms and contain compounds with established anti-inflammatory properties (31, 36). The majority of participants indicated that their use of these remedies was very helpful, consistent with the reported therapeutic efficacy documented in the ethnopharmacological literature cited above.

### 4.2 Thematic analysis findings

Five major themes emerged from the analysis, reflecting the variation and dynamics of traditional medicine responses across BRICS and SADC countries.

### 4.3 Theme 1: institutional support and integration variations

Stark differences emerged in formal support and integration of traditional medicine into health systems across countries (60).

Table 6 reveals stark hierarchical differences in how BRICS and SADC nations have institutionalized traditional medicine within formal health systems. China represents the pinnacle of formalization with state-sanctioned protocols, clinical trials, and government-funded research institutions that enabled Traditional Chinese Medicine (TCM) to be integrated into national pandemic guidelines, deployed in over 90% of confirmed COVID-19 cases. India occupies a middle position with AYUSH ministry guidance providing a structural framework, yet implementation remains fragmented across regions with limited documentation of clinical outcomes, reflecting governance complexity where multiple states pursue different integration pathways.

**Table 6:** Institutional support for IKS across countries.

Country	Level of formalization	Key features	Pandemic integration
China	Highest formalization	State-sanctioned protocols; clinical trials; government-funded research institutions	TCM included in national guidelines; used in >90% of confirmed cases
India	Moderate support	AYUSH ministry guidance; regional variation in implementation	Fragmented healthcare governance; limited documentation of outcomes
South Africa	Limited institutional backing	Traditional Health Practitioners Act (2007), but weak implementation	Traditional healers are largely excluded from pandemic planning
Zimbabwe	Largely unregulated	No formal integration framework	Traditional healers excluded from pandemic response

The stark contrast between BRICS and SADC reveals what might be termed “epistemic institutionalization.” China and India’s formalization contrasts sharply with SADC countries’ marginalization, reflecting broader patterns of health system development and political will. This is not merely a technical difference it reflects centuries of documented knowledge traditions versus oral systems. Whilst China and India benefit from centuries of documented traditions enabling research validation and policy integration, SADC countries’ oral traditions face regulatory obstacles despite deep knowledge bases. Standardization, often promoted as a solution to validate oral traditions, may be inappropriate as it reflects Western scientific epistemology; oral traditions represent different knowledge systems with their own validity claims.

South Africa presents a paradox: despite the Traditional Health Practitioners Act (2007), institutional backing remains limited, with traditional healers largely excluded from pandemic planning. Zimbabwe remains entirely unregulated with no formal integration framework, leaving traditional healers completely outside official pandemic responses. This exclusion is not accidental it reflects colonial legacies embedded in health governance structures.

### 4.4 Theme 2: documentation and preservation approaches

Table 7 crystallizes an epistemological divide: BRICS nations benefit from well-documented systems (Ayurveda, TCM) with over 1,000 clinical trials conducted since 2020, while SADC contexts rely predominantly on oral transmission with minimal research infrastructure and limited representation in global ethnopharmacology research.

**Table 7:** Documentation differences between BRICS and SADC.

Dimension	BRICS (China, India)	SADC (South Africa, Zimbabwe)
Documentation status	Well-documented systems (Ayurveda, TCM)	Predominantly oral transmission
Clinical trials (2020+)	Over 1,000 trials conducted	Minimal research infrastructure
Pharmaceutical contribution	~30% of modern pharmaceuticals are derived from IKS (33)	Few commercial products are derived from local traditions
Global research representation	Substantial	Limited representation in ethnopharmacology research

### 4.5 Theme 3: barriers to integration with biomedicine

This table masks a profound power asymmetry. Approximately 30% of modern pharmaceuticals are derived from IKS globally, yet few commercial products have emerged from SADC local traditions. This disparity reflects both differential research investment and the historical extraction of knowledge during colonial periods. African plants were systematized in European institutions, while African practitioners remained unrecognized and uncompensated (Table 8).

**Table 8:** Barriers to integration with biomedicine.

Barrier category	Specific barriers
Epistemological gaps	Holistic, spiritual, relational IKS vs. reductionist, mechanistic biomedical frameworks (2, 3)
Colonial legacies	Criminalisation policies; Witchcraft Suppression Act
Regulatory gaps	Absence of standardized pharmacopoeias and quality control (6, 29)
Resource constraints	Limited research funding and partnerships

The documentation difference has concrete consequences: documented systems create intellectual property regimes, clinical evidence hierarchies, and policy pathways that are inaccessible to oral traditions. Digital archiving and community-based documentation represent alternative preservation strategies respecting knowledge sovereignty. This suggests that the solution is not forcing oral knowledge into written academic formats, but rather creating hybrid documentation models that preserve knowledge within community control. The absence of representation in global ethnopharmacology research also means SADC IKS contributions remain invisible in international policy discourse. When global health bodies (WHO, UN, etc.) convene on traditional medicine, they predominantly cite BRICS-validated evidence, inadvertently rendering African knowledge systems peripheral despite their demonstrated utility during crises.

### 4.6 Theme 4: digital platforms and knowledge dissemination

Table 9 captures how WhatsApp emerged as the primary medium for IKS information sharing, democratizing access to knowledge previously confined to specific lineages while simultaneously enabling misinformation to circulate. Facebook facilitated broader community reach across geographic boundaries but allowed unverified claims to spread rapidly, while local radio remained accessible in low-connectivity areas but offered limited interactivity for verification.

**Table 9:** Digital platform usage for IKS information.

Platform	Key role	Benefits	Challenges
WhatsApp	Primary medium for sharing remedies	Democratized access to knowledge previously confined to specific lineages	Misinformation alongside validated information
Facebook	Community group discussions	Enabled broader reach across geographic boundaries	Unverified claims circulate rapidly
Local radio	Traditional medicine segments	Accessible in low-connectivity areas	Limited interactivity for verification

The digital platform data reveals a fundamental shift in knowledge governance. During the COVID-19 pandemic, the dissemination of traditional remedies via social media platforms such as WhatsApp and Facebook catalyzed a new wave of ethnobotanical engagement, both within and beyond indigenous communities. Whilst concerns remain about misinformation, these platforms democratized access to knowledge that was once restricted to specific lineages and oral networks. This democratization is simultaneously liberatory and threatening. On one hand, WhatsApp enabled rapid knowledge-sharing in crisis contexts where formal healthcare was inaccessible. On the other hand, the speed and reach of digital platforms cannot distinguish between validated traditional practices and dangerous misinformation, creating what might be termed “epistemic chaos”.

The table’s platform categories suggest a hierarchy of accessibility that mirrors socioeconomic inequality. WhatsApp dominance reflects smartphone penetration among middle-income users; radio accessibility indicates rural/low-connectivity populations were partially served but remain marginalized in digital discourse. This means pandemic preparedness policies that rely solely on digital dissemination will systematically exclude the most vulnerable populations. The role of digital platforms in both enabling and complicating knowledge dissemination demands careful attention; these tools can democratize access whilst also spreading misinformation, requiring media literacy and community engagement strategies. This demands active content curation, community fact-checking protocols, and integration of traditional healers as trusted information gatekeepers rather than passive consumers.

These three tables collectively demonstrate that IKS integration is fundamentally a governance problem, not merely a technical or evidential one. Institutional capacity (Table 6) determines whether knowledge systems are valued; documentation models (Table 7) determine whose knowledge counts as valid; and digital infrastructure (Table 9) determines whose voice reaches decision-makers during crises. Until SADC nations invest in institutional frameworks, alternative documentation pathways, and inclusive digital strategies, the marginalization of African IKS will persist despite demonstrated efficacy and community reliance.

### 4.7 Theme 5: community resilience and adaptability

Communities modified remedies according to symptoms and resource availability, facilitated intergenerational knowledge transmission, and pragmatically integrated traditional remedies with biomedical treatments when accessible (14, 15). These dynamics illustrate the critical role of local adaptation and knowledge custodianship in sustaining traditional medicine practices under pandemic pressures (16).

### 4.8 Safety and adverse effects

Eight participants (21.1% of the sample) reported minor adverse effects or remedy limitations: nausea (*n* = 3), stomach upset (*n* = 2), allergic skin reactions (*n* = 2), and diarrhea (*n* = 1). No serious adverse events were reported. This finding underscores that while traditional remedies are generally perceived as helpful by most participants, safety cannot be assumed, and individual responses vary substantially.

## 5 Discussion

### 5.1 Integration of findings with existing literature

This study contributes to growing evidence that Indigenous Knowledge Systems provide vital complementary roles in health system responses during global crises. The perceived effectiveness (85.2% self-reported benefit) aligns with previous studies demonstrating immune-supportive and antiviral properties of remedies used by participants (30, 35). *Artemisia afra*, *Lippia javanica*, and other remedies used have published evidence supporting efficacy against respiratory infections and immune dysfunction (13, 31). However, it is critical to note that this 85.2% figure represents participants’ subjective experience of symptom improvement, not clinical efficacy. Placebo effects, natural disease course, and concurrent biomedical treatments cannot be ruled out as contributors to reported improvement (60).

Our comparative findings on institutional integration align with WHO observations about traditional medicine policy variation globally (10, 65). China and India’s formalization contrasts sharply with SADC countries’ marginalization, reflecting broader patterns of health system development and political will (32).

### 5.2 Epistemological considerations and decolonial implications

A key contribution is explicit examination of epistemological differences between BRICS and SADC traditions (2, 53). Whilst China and India benefit from centuries of documented traditions enabling research validation and policy integration, SADC countries’ oral traditions face regulatory obstacles despite deep knowledge bases (1, 3).

Standardization, often promoted as a solution to validate oral traditions, may be inappropriate (3). Standardization reflects Western scientific epistemology; oral traditions represent different knowledge systems with their own validity claims (2). Decolonial approaches must recognize epistemological diversity rather than force oral knowledge into written frameworks (49).

Digital archiving and community-based documentation represent alternative preservation strategies respecting knowledge sovereignty (38). Technologies such as blockchain for intellectual property protection offer new possibilities for equitable benefit-sharing when IKS is commercialized (66).

### 5.3 How frameworks address each research question

Table 10 demonstrates that our dual theoretical frameworks planetary health and decoonial paradigms systematically address each research question, moving beyond descriptive reporting to critical analysis. RQ1 (remedy use) through the planetary health lens emphasizes ecosystem resources (local plants, herbs) and their sustainable harvesting, revealing that IKS mobilization is inseparable from environmental stewardship. Decolonially, the same RQ1 data validates non-biomedical conceptualizations of effectiveness participants’ holistic understanding of health improvement, spiritual dimensions, and relational healing without translating these into scientific reductionism.

**Table 10:** Framework contributions to research questions.

Research question	Planetary health contribution	Decolonial contribution
RQ1: Remedy use	Focus on ecosystem resources (herbs, plants) used in remedies	Validates non-biomedical conceptualizations of health and effectiveness
RQ2: Barriers	Examines environmental accessibility and resource sustainability	Identifies epistemic hierarchies and regulatory barriers rooted in colonial legacies
RQ3: Institutional differences	Analyzes health system integration as equity issue	Compares documented (BRICS) vs. oral (SADC) traditions without privileging either
RQ4: Digital platform**s**	Assesses digital access as environmental/social determinant	Centers community agency in knowledge dissemination and protection

RQ2 (barriers) is illuminated distinctly through each lens: planetary health reveals how environmental degradation and resource scarcity constrain remedy access, whilst decolonial analysis exposes epistemic hierarchies and colonial regulatory legacies that deliberately criminalize traditional practice. This dual analysis prevents mis-framing barriers as merely technical (lack of clinical evidence) when they are fundamentally political (marginalization rooted in colonial history).

RQ3 (institutional differences across BRICS vs. SADC) emerges as perhaps the most revealing through the combination of both frameworks. China’s integration of TCM within formal health systems reflects state recognition of IKS as complementary to biomedicine, yet risks co-optation and the subordination of traditional epistemologies to state control.

SADC countries’ exclusion of traditional healers reflects both lack of political will and active criminalization, representing what Table 10 identifies as epistemic hierarchies and regulatory barriers rooted in colonial legacies. Neither integration model is unproblematically” better” both involve power asymmetries requiring ongoing decolonial scrutiny.

RQ4 (digital platform role) through both lenses reveals platforms as dual-edged tools: they democratize access to knowledge previously confined to specific lineages and geographic networks, yet simultaneously enable misinformation and complicate verification. The planetary health lens emphasizes digital platforms as social and environmental determinants of health access; the decolonial lens centers community agency in knowledge dissemination and the risks of external actors controlling knowledge narratives through algorithm curation (Table 11).

**Table 11:** Policy implications and pandemic preparedness.

Recommendation	Target	Key actions
Formal recognition	National governments	Recognize traditional medicine as a complementary health modality in pandemic preparedness frameworks (65, 67, 70)
Research infrastructure	SADC region	Establish regional centres of excellence for traditional medicine research, validation, and documentation (24, 29)
Regulatory pathways	National regulators	Create regulatory pathways for traditional medicines distinct from pharmaceutical drugs (6)
Knowledge protection	International bodies	Implement robust mechanisms protecting traditional knowledge intellectual property and equitable benefit-sharing (66, 68)
Health workforce education	Academic institutions	Incorporate IKS competencies into medical and public health curricula (49, 52)
Inclusive planning	Government agencies	Include traditional healers, community leaders, and affected populations in pandemic preparedness planning (14, 15)

### 5.4 Policy implications and pandemic preparedness

The Policy Implications table outlines six key action areas; however, implementation requires substantially deeper institutional analysis than the table format permits. We elaborate each recommendation below with explicit attention to potential pitfalls and equity implications: Formal Recognition (National Governments): The recommendation to formally recognize traditional medicine as complementary in pandemic preparedness frameworks must be 13 accompanied by explicit protections against biomedical co-optation. Recognition without autonomy—where traditional healers become subordinated to biomedical authority— reproduces colonial hierarchies under a veneer of integration. SADC countries should reference WHO guidelines (65, 67) whilst resisting pressure to standardize or medicalize practices that may lose efficacy through reformulation. Formal recognition must include protection of traditional healers’ right to practice autonomously and their inclusion as decision-makers (not mere consultants) in pandemic planning. Research Infrastructure (SADC Region): Establishing regional centres of excellence for traditional medicine research requires sustained funding commitments and institutional autonomy. Critical here is resisting pressure to conduct only Western-style randomized controlled trials, which may be epistemologically inappropriate. Research infrastructure should include methods for documenting oral knowledge, supporting community-based research led by traditional practitioners themselves, and ensuring that research outputs benefit source communities through intellectual property protections and equitable benefit-sharing mechanisms (66, 68) (Table 11).

Regulatory Pathways (National Regulators): Creating separate regulatory pathways for traditional medicines distinct from pharmaceutical drugs acknowledges the different epistemologies and production processes involved. Overstandardization risks destroying the adaptive, contextual nature of IKS. Regulatory frameworks should permit variation, acknowledge environmental and seasonal influences on plant composition, and be co-designed with traditional healers rather than imposed by biomedical regulators. Knowledge Protection (International Bodies): Robust intellectual property protections and equitable benefit-sharing mechanisms are non-negotiable to prevent biopiracy and ensure source communities benefit when IKS knowledge is commercialized. This requires supporting traditional knowledge registries, protecting against patenting of traditional innovations by external actors, and establishing meaningful community consent protocols for commercialization.

Health Workforce Education (Academic Institutions): Incorporating IKS competencies into medical and public health curricula goes beyond teaching cultural sensitivity.” It requires fundamentally restructuring curricula to present IKS as epistemologically valid knowledge systems, not folklore. Medical students should learn alongside traditional practitioners as equals, not as passive recipients of biomedical experts’ explanations of what traditional medicine actually does.” Inclusive Planning (Government Agencies): Including traditional healers in pandemic preparedness planning demands dismantling criminalization laws (such as Zimbabwe’s and South Africa’s amended but problematic Witchcraft Suppression Acts) and recognizing traditional healers as legitimate members of essential workforces. Participation cannot be tokenistic consultation; it requires decision-making authority, resource allocation, and genuine power-sharing.

### 5.5 Synthesis: integrating gender, environmental, and equity dimensions

Beyond the specific policy recommendations, three cross-cutting dimensions demand urgent attention. First, gender dynamics remain insufficiently addressed in both research and policy. Women custodians of traditional knowledge remain systematically excluded from formal health decision-making, despite being primary knowledge keepers and healers in many communities (9, 14). Future pandemic preparedness must explicitly center women’s leadership, not merely their participation as community members.

Second, the environmental sustainability of IKS resource harvesting, whilst addressed through the planetary health framework in our analysis, remains underexamined in policy implementation. Increased demand for traditional remedies during health crises can drive unsustainable harvesting and species depletion. Policy frameworks must integrate environmental protection with IKS integration, requiring traditional knowledge about sustainable harvesting alongside biodiversity conservation.

Third, equity imperatives extend beyond the Global South. Within Global North countries, Indigenous populations and marginalized communities also rely on traditional and complementary medicine, yet face similar marginalization and regulatory barriers. The implications of this research thus apply transnationally, though with careful attention to local contexts and community-specific needs.

Despite these limitations and cautionary considerations, findings provide valuable exploratory insights into how communities mobilize indigenous knowledge during crises, the profound institutional variation in traditional medicine integration across global regions, and the specific policy gaps and structural reforms required for meaning.

## 6 Conclusion

This study demonstrated that Indigenous Knowledge Systems (IKS), particularly traditional medicine practices, played a critical and under-recognized role in community resilience during the COVID-19 pandemic across BRICS and SADC countries (15, 17). Despite facing systemic marginalization rooted in colonial legacies and biomedical dominance, African, Indian, and Chinese communities mobilized centuries of accumulated ethnobotanical and spiritual wisdom to address respiratory and immune health challenges (16, 18, 24).

The research reveals profound disparities in how different nations have approached IKS integration (28, 32). China and India have leveraged their documented traditions within formal health systems, whilst SADC countries, operating primarily with oral knowledge, have relied on informal community responses without institutional support (26, 36). These disparities are not inevitable; they reflect political choices and knowledge hierarchies that can be actively transformed through deliberate policy reform and investment (49, 53).

The findings underscore several critical imperatives (17, 24). First, IKS must be recognized not as a supplement to biomedical care but as an essential component of pluralistic, equitable, and culturally coherent health systems (22, 25). Second, the integration of IKS requires more than documentation; it demands structural reform in research funding, regulatory frameworks, and medical education to honor Indigenous epistemologies and ensure that traditional healers are full partners in health governance (1, 52). Third, the role of digital platforms in both enabling and complicating knowledge dissemination demands careful attention (38); these tools can democratize access whilst also spreading misinformation, requiring media literacy and community engagement strategies.

Moving forward, the SADC region must establish regional centres of excellence, harmonize policy frameworks across borders, and invest in collaborative research that bridges traditional healers, ethnobotanists, and biomedical scientists (24, 29). Gender dynamics must be centred, as women custodians of traditional knowledge remain systematically excluded from formal health decision-making (9, 14). Intellectual property protections and equitable benefit-sharing mechanisms must ensure that commercialisation of traditional remedies benefits source communities rather than external corporations (66, 68, 70).

This study ultimately argues for a decolonial, pluralistic approach to health that recognizes IKS not through the lens of Western scientific validation alone, but through the lived experiences, cultural coherence, and demonstrated effectiveness of Indigenous and traditional health practitioners (2, 49). The COVID-19 pandemic revealed both the fragility of biomedical-centric systems and the resilience embedded in local, culturally grounded health practices (16, 17). As we confront ongoing and emerging health threats within a context of ecological crisis and planetary health imperatives, the integration of Indigenous Knowledge Systems is not merely a matter of equity and cultural respect—it is a practical necessity for building robust, adaptive, and resilient health systems capable of serving all peoples (22, 24).

## Data Availability

The raw data supporting the conclusions of this article will be made available by the authors, without undue reservation.
